# Bioinspired Synthesis and Characterization of Dual-Function Zinc Oxide Nanoparticles from *Saccharopolyspora hirsuta*: Exploring Antimicrobial and Anticancer Activities

**DOI:** 10.3390/biomimetics9080456

**Published:** 2024-07-25

**Authors:** Essam N. Sholkamy, Mohamed A. A. Abdelhamid, Hazim O. Khalifa, Mi-Ran Ki, Seung Pil Pack

**Affiliations:** 1 Department of Botany and Microbiology, College of Science, King Saud University, P.O. Box 2455, Riyadh 11451, Saudi Arabia; elisi@ksu.edu.sa; 2 Department of Biotechnology and Bioinformatics, Korea University, Sejong-ro 2511, Sejong 30019, Republic of Korea; mohamed42@korea.ac.kr; 3 Department of Botany and Microbiology, Faculty of Science, Minia University, Minia 61519, Egypt; 4 Department of Veterinary Medicine, College of Agriculture and Veterinary Medicine, United Arab Emirates University, Al Ain P.O. Box 1555, United Arab Emirates; hazimkhalifa@uaeu.ac.ae; 5 Department of Pharmacology, Faculty of Veterinary Medicine, Kafrelsheikh University, Kafr El-Sheikh 33516, Egypt; 6 Institute of Industrial Technology, Korea University, Sejong-ro 2511, Sejong 30019, Republic of Korea

**Keywords:** bioinspired synthesis, *Saccharopolyspora hirsuta*, *Tapinoma simrothi*, zinc oxide nanoparticles, anticancer agent, antibacterial agent

## Abstract

Microbial synthesis offers a sustainable and eco-friendly approach for nanoparticle production. This study explores the biogenic synthesis of zinc oxide nanoparticles (ZnO-NPs) utilizing the actinomycete *Saccharopolyspora hirsuta* (Ess_amA6) isolated from *Tapinoma simrothi*. The biosynthesized ZnO-NPs were characterized using various techniques to confirm their formation and properties. UV–visible spectroscopy revealed a characteristic peak at 372 nm, indicative of ZnO-NPs. X-ray diffraction (XRD) analysis confirmed the crystalline structure of the ZnO-NPs as hexagonal wurtzite with a crystallite size of approximately 37.5 ± 13.60 nm. Transmission electron microscopy (TEM) analysis showed the presence of both spherical and roughly hexagonal ZnO nanoparticles in an agglomerated state with a diameter of approximately 44 nm. The biogenic ZnO-NPs exhibited promising biomedical potential. They demonstrated selective cytotoxic activity against human cancer cell lines, demonstrating higher efficacy against Hep-2 cells (IC50 = 73.01 µg/mL) compared to MCF-7 cells (IC50 = 112.74 µg/mL). Furthermore, the biosynthesized ZnO-NPs displayed broad-spectrum antimicrobial activity against both *Pseudomonas aeruginosa* and *Staphylococcus aureus* with clear zones of inhibition of 12.67 mm and 14.33 mm, respectively. The MIC and MBC values against *P. aeruginosa* and *S. aureus* ranged between 12.5 and 50 µg/mL. These findings suggest the potential of *S. hirsuta*-mediated ZnO-NPs as promising biocompatible nanomaterials with dual applications as antimicrobial and anticancer agents.

## 1. Introduction

Bionanotechnology leverages biological systems for the controlled synthesis of nanomaterials, offering a promising approach to sustainable nanomaterial production. This environmentally friendly method utilizes biological organisms, such as bacteria, for either the intracellular or extracellular fabrication of nanoparticles [1,2,3]. Biosynthesis offers a cost-effective and benign alternative to traditional synthetic methods that often rely on hazardous chemicals, high energy consumption, and complex procedures [4]. Biosynthesized nanoparticles exhibit enhanced properties compared to their bulk counterparts, including increased durability, higher surface area, and improved permeability [5,6]. These characteristics make them particularly attractive for various applications in diverse fields [7,8]. Given the limitations of conventional synthesis methods and the growing demand for sustainable nanomaterials, the development of green biosynthesis techniques holds significant promise for the future [9].

Actinobacteria, filamentous Gram-positive bacteria ubiquitous in marine and terrestrial ecosystems, represent a versatile resource for environmentally friendly nanomaterial production [10,11,12]. These microbes possess a remarkable biosynthetic repertoire, generating a diverse array of novel biologically active secondary metabolites with promising applications in biomedicine, including anticancer, antimicrobial, anti-aging, and anti-inflammatory properties [13,14,15]. Notably, actinobacteria establish symbiotic relationships with insects, enhancing their fitness in challenging environments [15,16]. Recent research highlights the potential of symbiotic actinobacteria as potent biofactories for the synthesis of metal nanoparticles (NPs) [17]. These bacteria produce unique secondary metabolites [18,19] and exopolysaccharides (EPSs) [20,21] that play a critical role in the biosynthesis process. Secondary metabolites act as effective reducing agents, facilitating the formation of NPs from metal ions. Additionally, EPSs influence the size, distribution, and morphology of the resulting nanoparticles, enabling a degree of control over the final product [22,23]. Studies indicate that actinobacteria-mediated NPs exhibit superior stability, dispersity, and biocidal properties, making them attractive candidates for various applications [24,25].

Zinc oxide nanoparticles (ZnO-NPs) have emerged as a particularly promising class of metal oxide NPs due to their advantageous properties, including non-toxicity, ease of synthesis, cost-effectiveness, wide bandgap, and high quantum efficiency [26,27]. Actinobacteria-mediated extracellular synthesis offers a sustainable approach for ZnO-NP production [28]. For example, soil-derived actinobacteria can be harnessed for the eco-friendly production of ZnO-NPs with antibacterial properties, as shown by their effectiveness against *Klebsiella pneumoniae* [29]. Additionally, marine *Streptomyces plicatus* emerges as a powerful tool for bioinspired synthesis of ZnO nanoparticles, boasting potent antibacterial and antibiofilm activities against *Streptococcus mutans* [28]. Similarly, ZnO-NPs produced with *Streptomyces* sp. displayed broad-spectrum antimicrobial activity against *Escherichia coli*, *Klebsiella pneumoniae*, *Bacillus subtilis, Staphylococcus aureus*, and *Candida fructus,* highlighting their potential application in antibacterial food packaging [30,31,32]. Beyond their well-established antimicrobial properties, ZnO-NPs have garnered significant attention for their diverse functionalities. Notably, recent research highlights their promising cytotoxic activity against a broad spectrum of cancer cell lines, including those associated with oral, breast, and cervical cancers [20,28]. This promising anticancer activity, potentially mediated by the generation of reactive oxygen species (ROS) within cancer cells, opens exciting avenues for the development of novel cancer treatment strategies [33].

Given the diverse functionalities of ZnO-NPs, particularly their potential applications in anticancer therapy and antimicrobial treatments, this study explores a green synthesis method for ZnO-NPs utilizing a novel actinobacterial strain, *Saccharopolyspora hirsuta* (ess_amA6), isolated from the insect *Tapinoma simrothi*. The study aims to characterize the biosynthesized ZnO-NPs, focusing on physicochemical properties relevant to their potential applications. Additionally, the study will evaluate the in vitro cytotoxic potential of the ZnO-NPs against various cancer cell lines, alongside their antimicrobial activity against *Staphylococcus aureus* and *Pseudomonas aeruginosa*.

## 2. Materials and Methods

### 2.1. Bacterial Strains

*Saccharopolyspora hirsuta* (ess_amA6), an actinobacterial strain isolated from the insect *Tapinoma simrothi* (GenBank accession no. KF996506) [34], was employed for the biosynthesis of ZnO-NPs. *Staphylococcus aureus* (ATCC 25923) and *Pseudomonas aeruginosa* (ATCC 27853), both kindly provided by the Bacteriological Laboratory, College of Science, King Saud University, were used to assess the antibacterial activity of the synthesized ZnO-NPs.

### 2.2. Biosynthesis of Zinc Oxide Nanoparticles

The culture of *S. hirsuta* (ess_amA6) was inoculated in starch casein nitrate broth and incubated at 30 °C for 4–5 days. After incubation, 5 mL of the *S. hirsuta* culture filtrate was added to a 95 mL solution of 0.1 M ZnSO_4_ in a 250 mL Erlenmeyer flask. The pH of the mixture (filtrate + 0.1 M ZnSO_4_) was adjusted to 8 using 0.4 N NaOH solution. The flask was kept in an incubator shaker (LABTECH INSTRUMENTS, Navi Mumbai, India) for 15 min at 40 °C with agitation at 120 rpm. Following incubation, the solution was cooled using ice and further incubated at 40 °C for 2 min. To allow for the settling of the synthesized nanoparticles due to colloidal solution formation, the mixture was centrifuged at 3000 rpm for 10 min. The resulting ZnO-NP pellets were collected and washed with deionized water to remove residual media components, and the supernatant was discarded. Finally, collected precipitates were dried overnight in a hot air oven (BMT USA, Monroe, WA, USA) at 80 °C to yield the ZnO-NPs [35].

### 2.3. Characterization of Zinc Oxide Nanoparticles

A comprehensive physicochemical characterization of the biosynthesized ZnO-NPs was performed using a variety of techniques. The UV–visible absorption spectrum of ZnO-NPs suspended in deionized water was acquired between 300 and 600 nm using UV–visible spectroscopy. This analysis sheds light on the optical properties of the nanoparticles. X-ray diffraction (XRD) analysis was employed to investigate the crystallinity of the synthesized ZnO-NPs. The Debye–Scherrer equation was subsequently utilized to calculate the average particle size. Functional group analysis of the ZnO-NPs was performed using Fourier Transform Infrared Spectroscopy (FTIR). Here, the ZnO-NPs were pelletized with KBr discs and scanned within the range of 500–4000 cm^−1^. Transmission electron microscopy (TEM) operated at 100 kV (TEM-JEOL-JEM1011, Tokyo, Japan) was used to determine the morphology of the ZnO-NPs. Complementary EDX analysis, performed in tandem with TEM, offered a comprehensive characterization of the nanoparticles’ elemental composition.

### 2.4. Determination of ZnO-NP Cytotoxic Activity

The in vitro cytotoxic potential of the biosynthesized ZnO-NPs against human cancer cell lines was assessed using the well-established MTT assay [36]. Two established cancer cell lines, i.e., breast adenocarcinoma (MCF-7) and hepatocellular carcinoma (Hep-2), were employed. Cells were individually seeded in 96-well plates and allowed to adhere for a predetermined time. Subsequently, they were exposed to a range of ZnO-NP concentrations (1, 5, 10, 25, 50, and 100 μg/mL) for 24 h. Following incubation, the culture medium was discarded, and the cells were subjected to a PBS wash to eliminate any residual nanoparticles. The MTT assay served to quantify cellular metabolic activity, a proxy for cell viability. Briefly, following incubation with MTT solution, viable cells metabolized the tetrazolium salt into formazan crystals. These crystals were subsequently dissolved in dimethyl sulfoxide (DMSO-Wanhua Chemical Group Co., Ltd., Yantai, China), and the resulting solution’s absorbance was measured at 550 nm. Cell viability was then expressed as a percentage relative to untreated controls, reflecting the potential cytotoxicity of ZnO-NPs.

### 2.5. Morphological Analysis of ZnO-NP-Treated Cancer Cells

MCF-7 and Hep-2 cells were individually cultured in 35 mm sterile Petri dishes at 37 °C in a CO_2_ incubator. Following adherence, cells were exposed to various concentrations of ZnO-NPs (both below and above their IC_50_ values) to evaluate their impact on cell morphology. The effects were then compared to untreated control cells using an inverted light microscope (Labomed, Los Angeles, CA, USA) [37].

### 2.6. Antibacterial Activity and Minimum Inhibitory/Bactericidal Concentrations (MIC/MBC) of ZnO-NPs

The antibacterial activity of biosynthesized ZnO-NPs against *Pseudomonas aeruginosa* and *Staphylococcus aureus* was evaluated using the agar well diffusion assay. Briefly, bacterial suspensions (adjusted to 0.5 McFarland standard) were spread onto sterile nutrient agar plates. After drying for 15 min, wells were created and loaded with 30 µg/mL ZnO-NP solution. Amoxicillin, a commonly used antibiotic, served as a positive control. Following incubation at 37 °C for 24 h, the diameters of the inhibition zones surrounding each well were measured [38]. The minimum inhibitory concentration (MIC) and minimum bactericidal concentration (MBC) of ZnO-NPs were determined using the microdilution method in nutrient broth. Serial two-fold dilutions of the bacterial cultures were prepared in broth, resulting in a concentration range of 100 to 0.75 µg/mL. Aliquots (100 µL) of each dilution were dispensed into a 96-well plate alongside growth controls (containing culture broth and bacteria) and sterility controls (containing only broth and no bacteria). All wells were inoculated with 10 µL of bacterial suspension (10^8^ CFU/mL). The microdilution plates were incubated for 24 h at 37 °C. Following incubation, a small volume from each well was subcultured onto fresh agar plates and incubated again at 37 °C for 24 h. The MIC was defined as the lowest concentration of ZnO-NPs that resulted in no visually detectable growth of bacteria on the subcultured plates, indicating the inhibition of bacterial growth. The MBC was defined as the lowest concentration that resulted in no bacterial growth after subculturing, indicating bactericidal activity. The MIC and MBC values were expressed in µg/mL.

### 2.7. Statistical Analysis

To ensure robust data analysis, all experiments were performed in triplicate following a fully randomized design. The data are presented as mean ± standard deviation (SD) to provide a comprehensive representation of the results and their variability. Statistical analysis was conducted using GraphPad Prism software (version 8; GraphPad Software, La Jolla, CA, USA) for all experiments. One-way analysis of variance (ANOVA) was employed to identify statistically significant differences between the experimental groups. Following a significant ANOVA result (*p* < 0.05), Tukey’s multiple comparison test was performed for post hoc analysis to pinpoint specific group differences. Throughout the study, a significance level of α = 0.05 was used to define statistically significant results.

## 3. Results and Discussion

### 3.1. Characterization of Biogenically Synthesized ZnO-NPs Using S. hirsute

Building upon the advantages of microbial biosynthesis, such as its eco-friendly nature, scalability, and tunable properties [18], this study explored the biogenic synthesis of ZnO-NPs using the actinobacterium *S. hirsuta*. The formation of ZnO-NPs was initially confirmed by the visual observation of a white precipitate within the starch casein nitrate broth, aligning with previous reports of white precipitate formation during ZnO-NP synthesis using *Sphingobacterium thalpophilum* [21] and *Lactobacillus sporangens* [39]. This initial observation, while suggestive, necessitated further confirmation through advanced analytical techniques to establish the successful synthesis and detailed structural properties of the nanoparticles.

**Figure 1:**
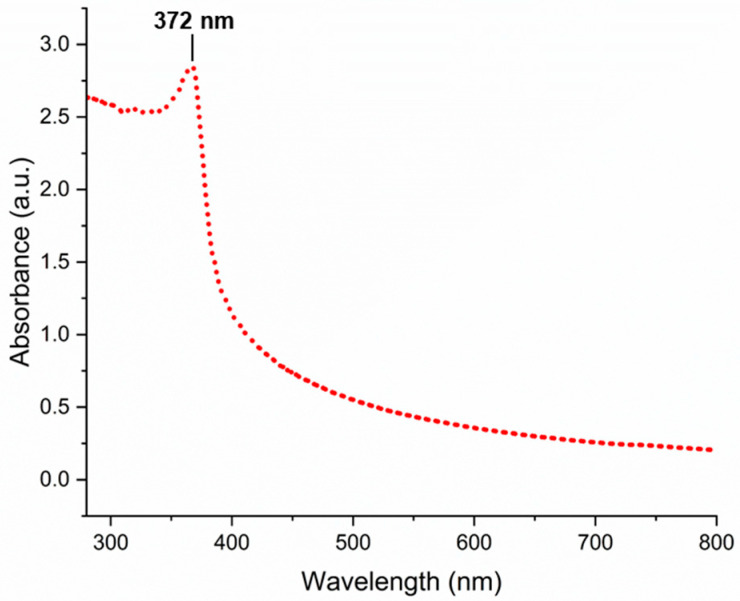
UV–visible absorption spectrum of biogenic *S. hirusta*-mediated ZnO nanoparticles.

UV–visible spectroscopy analysis provided further confirmation of ZnO-NP biosynthesis. The characteristic absorption peak observed at 372 nm (Figure 1) in the UV–visible spectrum is attributed to the intrinsic bandgap absorption of ZnO-NPs. This observation aligns well with previous studies, where ZnO-NPs synthesized using *Serratia nematodiphila* exhibited an absorption peak around 379 nm [40]. The specific wavelength of the absorption peak is related to the bandgap energy of ZnO-NPs, which can be influenced by factors such as particle size, shape, and presence of defects [41,42]. Additionally, the variations in the peak position can also be influenced by the choice of metal precursor and synthesis conditions [41]. These data suggest that the biogenic synthesis method employed with *S. hirsuta* can effectively produce ZnO-NPs with desirable optical properties.

**Figure 2:**
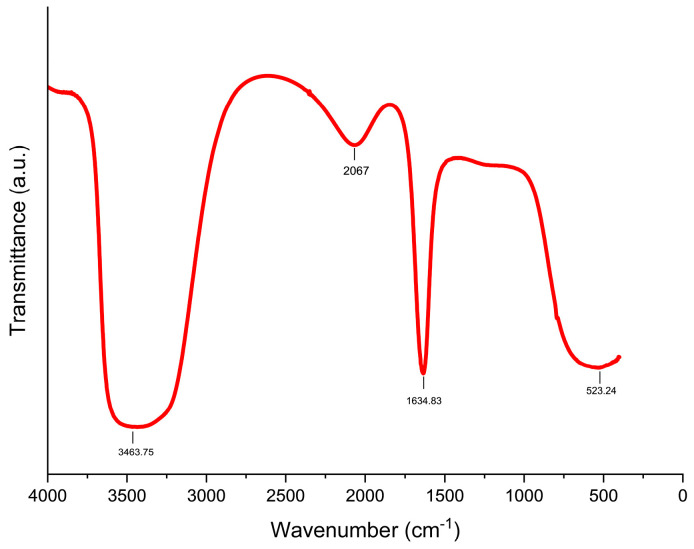
FTIR spectrum of ZnO nanoparticles biosynthesized using *S. hirsuta*.

The FTIR spectrum (Figure 2) of the biosynthesized ZnO-NPs revealed characteristic peaks at 3463.75 cm^−1^, 2067.01 cm^−1^, 1634.83 cm^−1^, and 523.24 cm^−1^ (Figure 2). These peaks provide insights into the chemical composition and the role of biomolecules in the synthesis process. The peak at 523.24 cm^−1^ corresponds to the stretching vibrations of Zn-O bonds, confirming the successful incorporation of zinc into the nanoparticle structure [43]. The peaks observed at 3463.75 cm^−1^ and 1634.83 cm^−1^ can be attributed to O-H stretching and bending signals of water molecules adsorbed on the ZnO surface and C=C stretching vibrations in alkenes, respectively, potentially originating from biomolecules associated with *S. hirsuta* during the synthesis process [44]. The presence of these functional groups is further supported by the broad peak at 2067.01 cm^−1^, indicative of carbonyl groups on the ZnO-NP surface [45]. The presence of these functional groups suggests that biomolecules from *S. hirsuta* not only facilitate the synthesis but also stabilize the ZnO-NPs. Previous studies have shown that such biomolecules, particularly those containing carbonyl and hydroxyl groups, enhance the stability of ZnO-NPs by preventing agglomeration and providing a capping effect [46,47]. This biogenic synthesis method thus leverages natural biomolecules to produce stable and functional ZnO-NPs, offering an eco-friendly alternative to chemical stabilizers.

**Figure 3:**
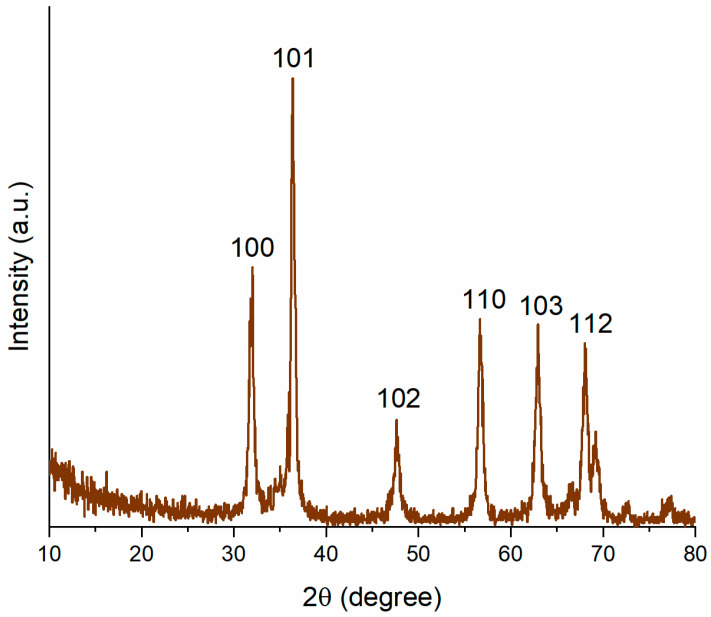
X-ray diffraction (XRD) pattern of *S. hirsuta*-mediated ZnO nanoparticles.

The XRD pattern of *S. hirsuta*-mediated ZnO-NPs (Figure 3) displayed distinct diffraction peaks at 2θ values of 31.74°, 36.20°, 47.45°, 56.55°, 62.75°, and 67.93° (Figure 3). These peaks correspond to the crystal planes with Miller indices (100), (101), (102), (110), (103), and (112), respectively. Notably, this observed pattern aligns well with the standard reference pattern for hexagonal wurtzite-structured ZnO (powder diffraction card no: 36-1451), unequivocally confirming the formation of ZnO-NPs with this specific crystal structure [48]. Furthermore, the observed broadening of diffraction peaks indicates the nanocrystalline nature of the synthesized material.

The Debye–Scherrer equation given below was utilized to estimate the average crystallite size (D) of the biogenic ZnO-NPs:

[D = kλ/(β cosθ)](1)

where D is the crystallite size (nm) of biosynthesized ZnO-NPs, k is Scherrer constant given by 0.94, λ is the wavelength of X-rays (CuKα radiation, 1.541 Å), θ is the Bragg diffraction angle, and β is the full-width at half-maximum (FWHM) of the diffraction peak corresponding to a plane (101).

The Scherrer equation (Equation (1)) was employed to estimate the average crystallite size of the biogenic ZnO-NPs based on the most intense peak (β = 0.2533) located at 2θ = 36.20° in the XRD pattern. This peak corresponds to the (101) crystal plane of ZnO. The calculated average crystallite size using this method was approximately 37.48 nm. The minor diffraction peaks observed in the XRD spectrum could be attributed to the crystallization of secondary metabolites or other biomolecules adsorbed onto the ZnO-NP surface, which is a characteristic feature of biogenically synthesized nanoparticles [49].

**Figure 4:**
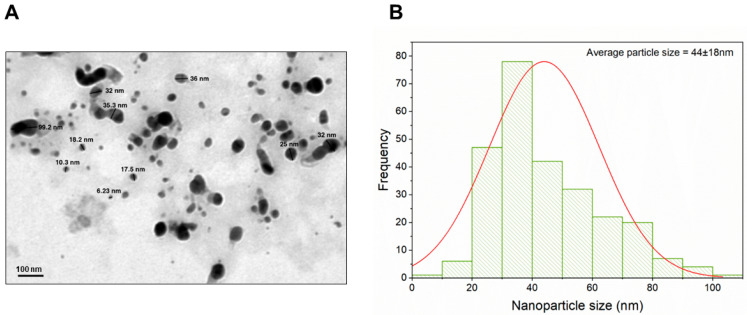
(**A**)Transmission electron microscopy (TEM) analysis of ZnO nanoparticles biosynthesized using *S. hirsuta* and (**B**) their corresponding particle size distribution histogram. The red line represents a Gaussian fit.

TEM analysis (Figure 4A) revealed that the *S. hirsuta*-mediated ZnO-NPs exhibited a mixed morphology, consisting of both spherical and hexagonal nanoparticles with some degree of agglomeration. The observed particle size distribution ranged from 0 to 90 nm (Figure 4B), indicating polydispersity within the sample. This polydispersity is commonly observed in biogenic nanoparticle synthesis and can be attributed to various factors during the biosynthesis process, such as precursor concentration, temperature, and incubation time. The mixed morphology and size distribution are consistent with previous reports on biogenic ZnO-NPs, highlighting the complexity and variability inherent in microbial synthesis methods [50].

**Figure 5:**
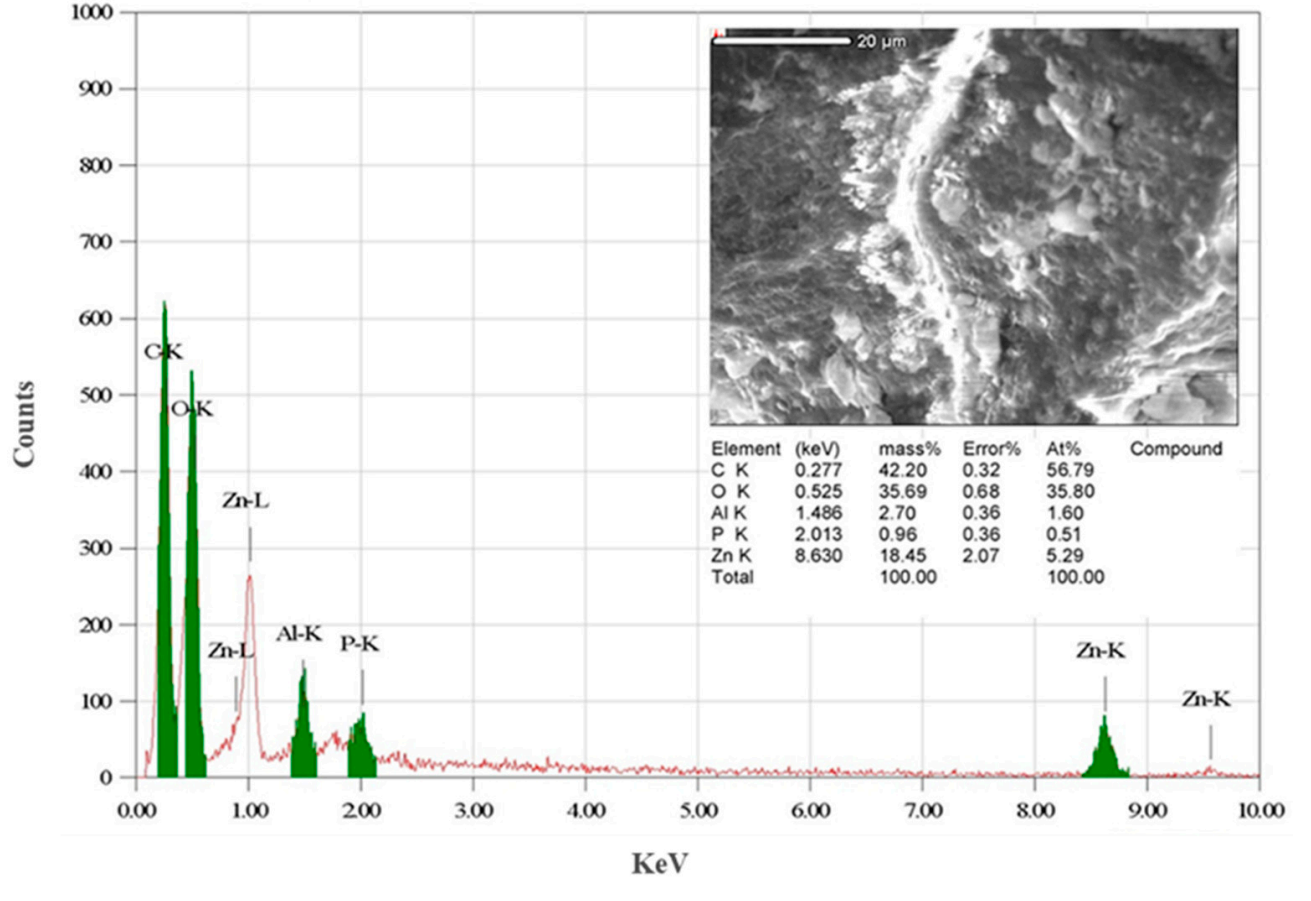
EDX analysis of *S. hirsuta*-mediated ZnO nanoparticles. The red and green lines represent the elemental composition of the zinc oxide nanoparticles.

EDX analysis (Figure 5) confirmed the presence of zinc (Zn), oxygen (O), and carbon (C) in the biosynthesized ZnO-NPs. The quantitative analysis indicated that the NPs were composed of approximately 42.2% carbon, 18.45% zinc, and 35.69% oxygen. The relatively high carbon content suggests the presence of organic surface moieties, potentially originating from biomolecules associated with *S. hirsuta* during the synthesis. This is in line with previous studies where biogenic ZnO-NPs exhibited significant carbon content due to the organic capping agents provided by the microbial biomass, which aid in nanoparticle stabilization and functionalization [51].

### 3.2. Evaluation of Biomedical Potential of S. hirsuta-Mediated ZnO-NPs

#### 3.2.1. Anticancer Potential

Conventional cancer treatment modalities often suffer from significant side effects, prompting the exploration of novel therapeutic strategies. Nanodrug delivery systems hold promise for cancer therapy due to their enhanced biocompatibility, potential for targeted drug delivery via surface functionalization, and the possibility of reduced side effects. ZnO-NPs have emerged as promising candidates in this arena, with several studies demonstrating their efficacy against various cancers [49,52,53,54,55]. The proposed mechanisms of action for ZnO-NP-mediated cytotoxicity include the generation of reactive oxygen species (ROS) within cancer cells, leading to apoptosis, cell cycle arrest, necrosis, and membrane damage [56]. This ROS production can also indirectly impact cellular DNA. Additionally, the nanoscale size of ZnO-NPs may facilitate their penetration into the nucleus of cancer cells, potentially enabling interaction with genetic material [57]. Furthermore, their high biocompatibility makes them attractive candidates for various therapeutic applications [58]. Notably, the enhanced solubility of ZnO-NPs compared to other nanoparticles may contribute to their amplified cytotoxic effects on diverse cancer cell lines [59].

**Figure 6:**
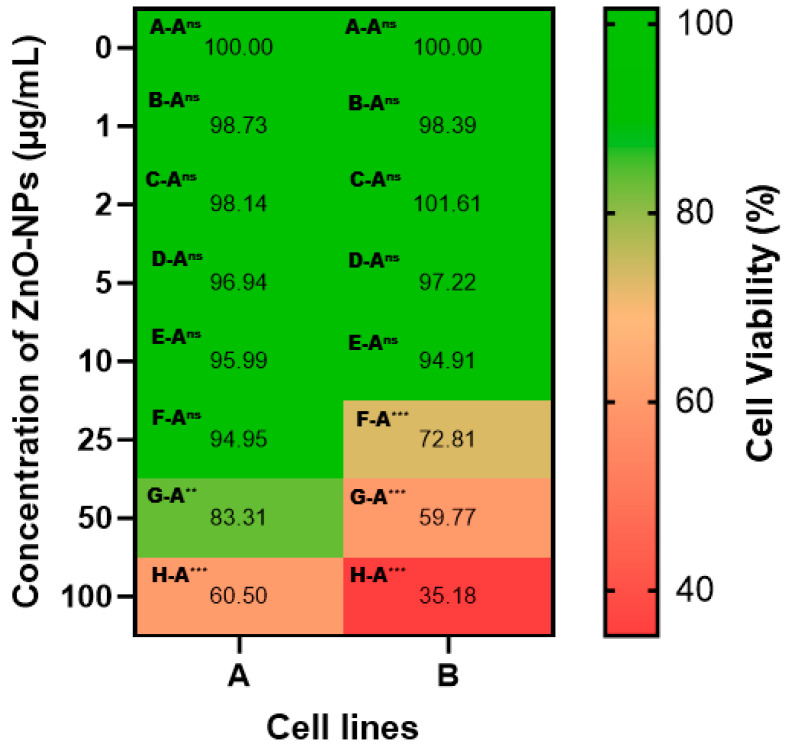
Cytotoxicity of *S. hirsuta*-mediated ZnO-NPs against human cancer cell lines. (**A**) MCF-7 cells and (**B**) Hep-2 cells. Data represent cell viability (%) after treatment with various concentrations of ZnO-NPs. Statistical significance is indicated: ** means *p* < 0.005, *** means *p* < 0.001, and ns means non-significance.

Next, we employed the MTT assay to evaluate the cytotoxic potential of *S. hirsuta*-mediated ZnO-NPs against MCF-7 and Hep-2 cancer cell lines. Both cell lines exhibited a significant dose-dependent decrease in cell viability upon ZnO-NP treatment. MCF-7 cells displayed a maximum viability of approximately 60.5% at the highest tested concentration of ZnO-NPs (100 µg/mL). Similarly, Hep-2 cell viability was reduced to around 35.2% at the same concentration (Figure 6). These findings suggest a potentially greater efficacy of *S. hirsuta*-mediated ZnO-NPs against Hep-2 cells compared to MCF-7 cells. The calculated IC50 values, representing the concentration required to inhibit cell viability by 50%, were 73.01 µg/mL and 112.74 µg/mL for Hep-2 and MCF-7 cells, respectively. Notably, previous studies have reported similar cytotoxic activities of synthesized ZnO-NPs against MCF-7 [60] and Hep-2 [61] cell lines, corroborating our observations.

**Figure 7:**
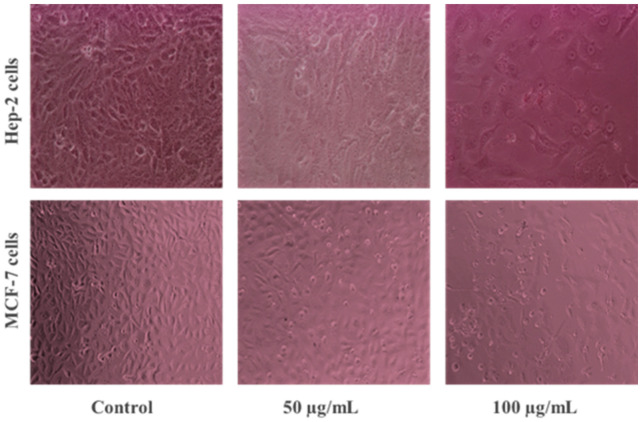
Morphological changes in cancer cells (Hep-2 and MCF-7) treated with *S. hirsuta*-mediated ZnO-NPs.

To assess the impact of *S. hirsuta*-mediated ZnO-NPs on cellular morphology, MCF-7 and Hep-2 cells were treated with varying concentrations encompassing the established IC50 values (Figure 7). This approach revealed dose-dependent morphological changes in both cell lines following a 24 h exposure to ZnO-NPs (0, 50, and 100 µg/mL). Treated cells exhibited shrinkage, detachment from the culture surface, distorted cell shapes, and the formation of blebs, contrasting with the homogenous, even, and uniform adherence pattern observed in untreated control cells. These morphological alterations are hallmarks of cellular stress and are consistent with the antiproliferative effects of ZnO-NPs reported in previous studies [61,62]. We further conducted a comparative analysis of cytotoxic activity. A prior study reported a 43% cytotoxicity in Hep-2 cells exposed to a lower concentration (20 µg/mL) of synthetic ZnO-NPs for 24 h [61]. In contrast, our findings demonstrate a higher cytotoxic activity (64.82%) for *S. hirsuta*-mediated ZnO-NPs simultaneously. This discrepancy might be due, in part, to the biogenic nature of our ZnO-NPs. Preliminary screening results revealed the S. hirsuta filtrate exhibited varying degrees of anticancer activity against MCF-7 and Hep-2 cell lines. This suggests inherent bioactivity within the filtrate, with surface-associated secondary metabolites potentially contributing a synergistic cytotoxic effect with the ZnO core.

However, the presence of a carbon-based capping layer on the biogenic ZnO nanoparticles necessitates further investigation. This capping layer could potentially impede the anticancer activity of the ZnO nanoparticles, highlighting the importance of exploring methods for their removal to optimize their therapeutic potential. These findings suggest a complex interplay between the ZnO nanoparticles, the inherent bioactivity of the *S. hirsuta* filtrate, and the capping layer. Further research is warranted to fully elucidate this interplay and potentially enhance the observed cytotoxic effects.

#### 3.2.2. Antimicrobial Potential

**Figure 8:**
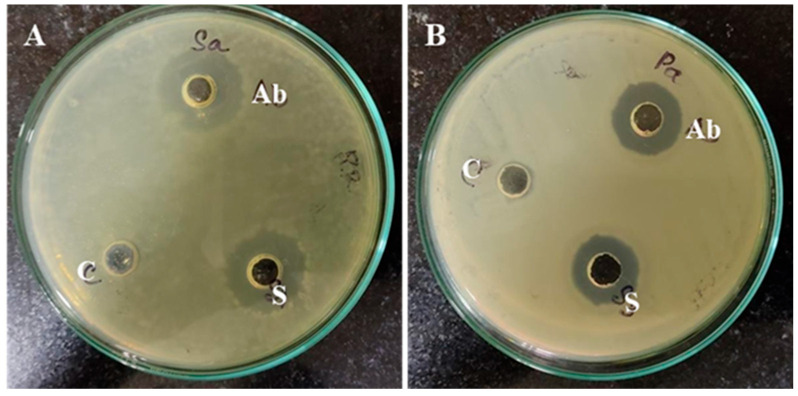
Antimicrobial activity of *S. hirsuta*-mediated ZnO-NPs (30 µg/mL) against (**A**) *S. aureus* and (**B**) *P. aeruginosa*. Treatments: C (control), Ab (antibiotic), and S (ZnO-NPs).

**Figure 9:**
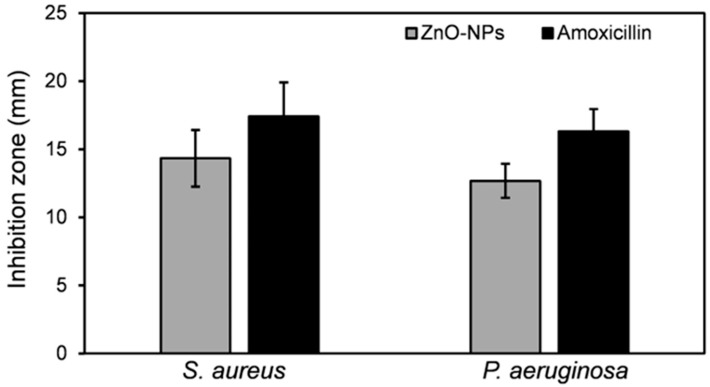
The growth inhibition zones of *S. aureus* and *P. aeruginosa* treated with *S. hirsuta*-mediated ZnO-NPs compared to amoxicillin (20 µg/mL).

The emergence of bacterial resistance to conventional antibiotics necessitates the exploration of novel antimicrobial agents [63,64]. Biogenic nanoparticles (NPs) derived from metal oxides, such as ZnO-NPs, have garnered considerable attention as promising alternatives due to their reported biocompatibility and non-toxic properties [65]. This study evaluated the antibacterial potential of *S. hirsuta*-mediated ZnO-NPs against *S. aureus* and *P. aeruginosa*, two prominent nosocomial pathogens. The findings revealed a statistically significant zone of inhibition for *S. aureus* (14.33 ± 2.52 mm) compared to *P. aeruginosa* (12.67 ± 2.08 mm) as depicted in Figure 8 and Figure 9. This observation suggests a greater susceptibility of *S. aureus* to the biogenic ZnO-NPs. The difference in susceptibility can be attributed to the structural differences in their cell walls; Gram-positive bacteria like *S. aureus* have a thicker peptidoglycan layer, which might be more susceptible to nanoparticle penetration and subsequent antibacterial action [66].

The antibacterial activity of nanoparticles (NPs) is attributed to several well-defined mechanisms. ZnO-NPs, in particular, exert their antibacterial effects through direct interactions with bacterial cell membranes. This disrupts membrane integrity and permeability, leading to the efflux of vital cellular components like nutrients and waste products, ultimately compromising bacterial survival [67]. Furthermore, ZnO-NPs can accumulate within the cytoplasm, where they interfere with critical metabolic processes by binding to and inhibiting the activity of essential proteins and enzymes [68]. Moreover, the dissolution of ZnO-NPs releases zinc ions (Zn^2+^) that can penetrate bacterial cells. These ions disrupt enzyme function by binding to thiol groups in proteins, further compromising cellular viability [69].

Notably, the existing literature reports that biogenic ZnO-NPs can exhibit double the efficacy compared to their chemically synthesized counterparts [35]. Interestingly, our preliminary data reveal moderate antibacterial activity of the *S. hirsuta* filtrate alone, reaching approximately 30–45% of the effect observed with biogenic ZnO-NPs. Considering that *S. hirsuta* belongs to the actinomycete phylum, well-known for producing potent antibacterial compounds [70], these findings suggest a potential synergistic interaction between the filtrate and ZnO-NPs. This synergy could be a key factor contributing to the enhanced antibacterial efficacy observed in our biogenic ZnO-NPs. Previous studies have successfully isolated such compounds from various actinomycete species, demonstrating their effectiveness against a wide range of bacteria. The bioactive compounds isolated from marine actinomycetes using ethyl acetate effectively inhibited multi-drug-resistant *S. aureus* [38]. Similarly, the *Streptomyces* strains isolated from soil samples produced bioactive compounds with antibacterial activity against *P. aeruginosa* [71]. Therefore, the enhanced antibacterial activity observed in *S. hirsuta*-mediated ZnO-NPs could be attributed to a synergistic interaction between ZnO and the bioactive metabolites produced by *S. hirsuta*.

**Figure 10:**
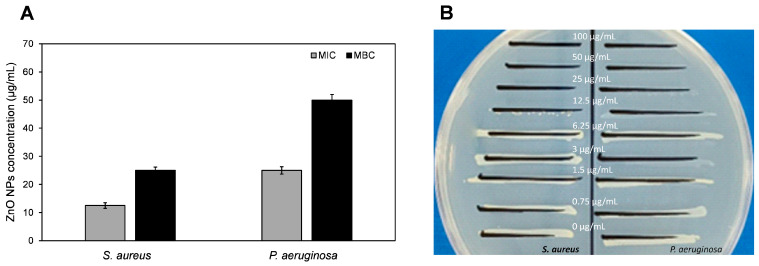
Antibacterial activity of *S. hirsuta*-mediated ZnO-NPs. (**A**) MIC and MBC values of ZnO-NPs against *S. aureus* and *P. aeruginosa*. (**B**) Representative images of bacterial subcultures following treatment with various concentrations of biogenic ZnO-NPs.

Interestingly, the *S. hirsuta*-mediated ZnO-NPs were more effective against the Gram-positive *S. aureus* (MIC: 12.5 μg/mL; MBC: 25 μg/mL) than the Gram-negative *P. aeruginosa* (MIC: 25 μg/mL; MBC: 50 μg/mL) (Figure 10A,B). This differential activity can be attributed to the structural differences in the cell walls of Gram-positive and Gram-negative bacteria. The outer membrane of Gram-negative bacteria like *P. aeruginosa* acts as a barrier to nanoparticles, thereby reducing their efficacy [66]. These findings suggest the potential of biosynthesized *S. hirsuta*-mediated ZnO-NPs as an alternative antimicrobial agent to combat drug-resistant bacterial infections. The enhanced antibacterial activity against *S. aureus* highlights their promise in treating infections caused by Gram-positive bacteria.

## 4. Conclusions

The growing interest in biogenic synthesis methods for nanomaterials has prompted us to explore natural resources like microbes for environmentally benign and sustainable nanoparticle production. In this study, we successfully demonstrated a facile method for synthesizing metal oxide NPs utilizing the extracellular fluid of *S. hirsuta* isolated from *T. simrothi*. The biogenic ZnO-NPs were comprehensively characterized using various techniques, confirming their hexagonal spherical morphology. Notably, these NPs exhibited significant cytotoxic activity against MCF-7 and HepG-2 cell lines, representatives of breast and hepatocellular carcinoma, respectively. Furthermore, the *S. hirsuta*-mediated ZnO-NPs displayed effective bactericidal activity against both *P. aeruginosa* and *S. aureus*, demonstrating broad-spectrum antimicrobial potential. These findings collectively highlight the promise of *S. hirsuta*-mediated ZnO-NPs as broad-spectrum agents with dual efficacy against both cancer and bacterial pathogens. Future investigations should delve deeper into the mechanisms of action underlying these observed effects. We are particularly interested in exploring the role of *S. hirsuta*-derived ZnO-NPs in stimulating reactive oxygen species (ROS) production and the subsequent consequences of elevated intracellular ROS levels in MCF-7 and HepG-2 cells. Elucidating these mechanisms is paramount for advancing this biogenic nanoformulation as a potential therapeutic agent in cancer treatment strategies.

## Data Availability

Data are contained within the article.
